# Supplementary material for: Actin polymerization promotes invagination of flat clathrin-coated lattices in mammalian cells by pushing at lattice edges
Source: Nat Commun. 2022 Oct 17;13:6127. doi: 10.1038/s41467-022-33852-2 (PMC9576739; doi:10.1038/s41467-022-33852-2)
Supplement: Supplementary file 1 — Supplementary Information [file 41467_2022_33852_MOESM1_ESM.pdf]

SUPPLEMENTARY INFORMATION

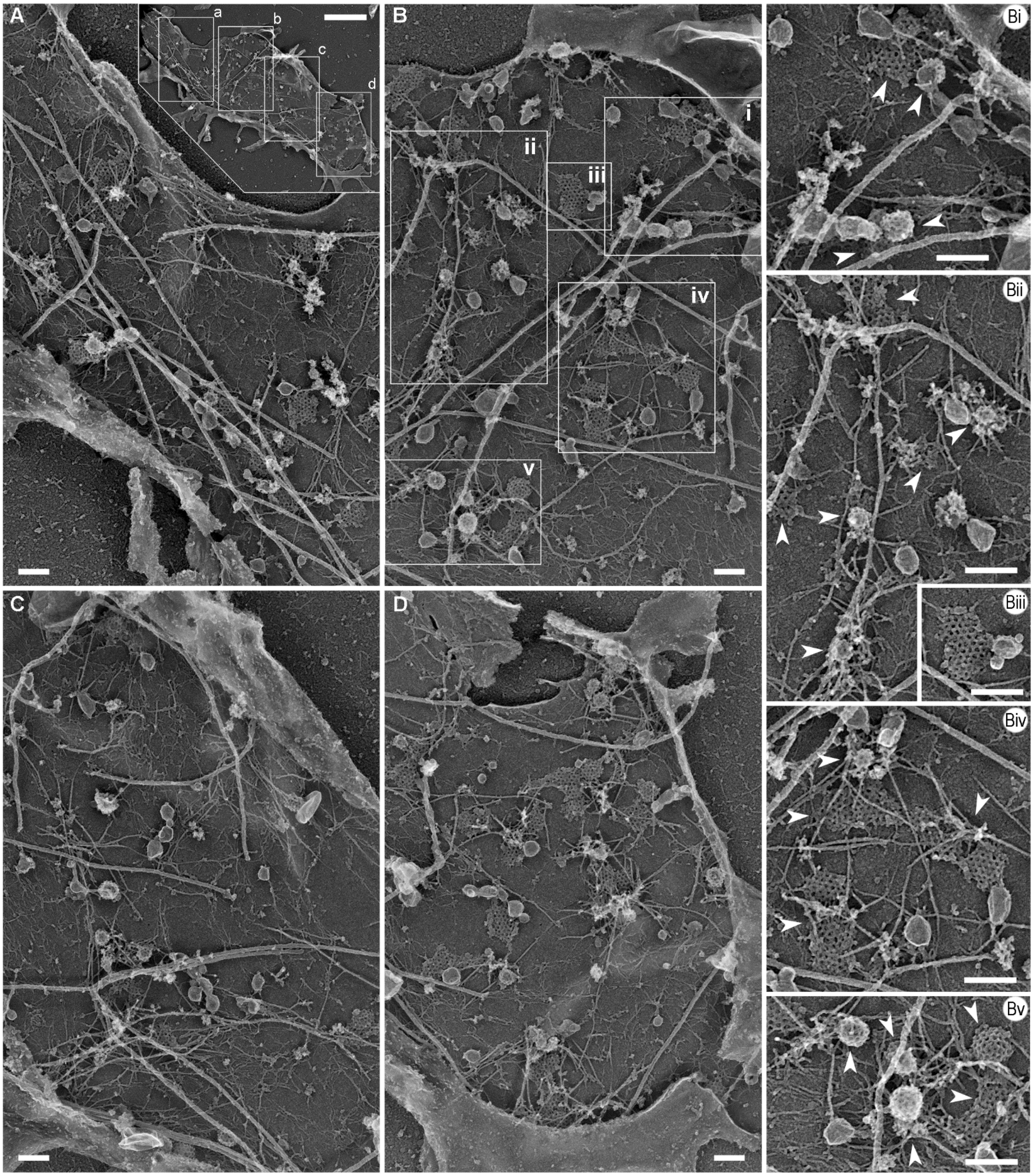

Supplementary Figure 1. CCS morphology in U2OS cells. (A, inset) PREM image of an entire plasma membrane sheet from an unroofed cell. (A-D) Enlarged boxed regions from the inset in A, which collectively cover almost entire plasma membrane sheet. (Bi – Bv) Enlarged boxed regions in B showing every CCS present in B. Arrowheads mark CCSs.

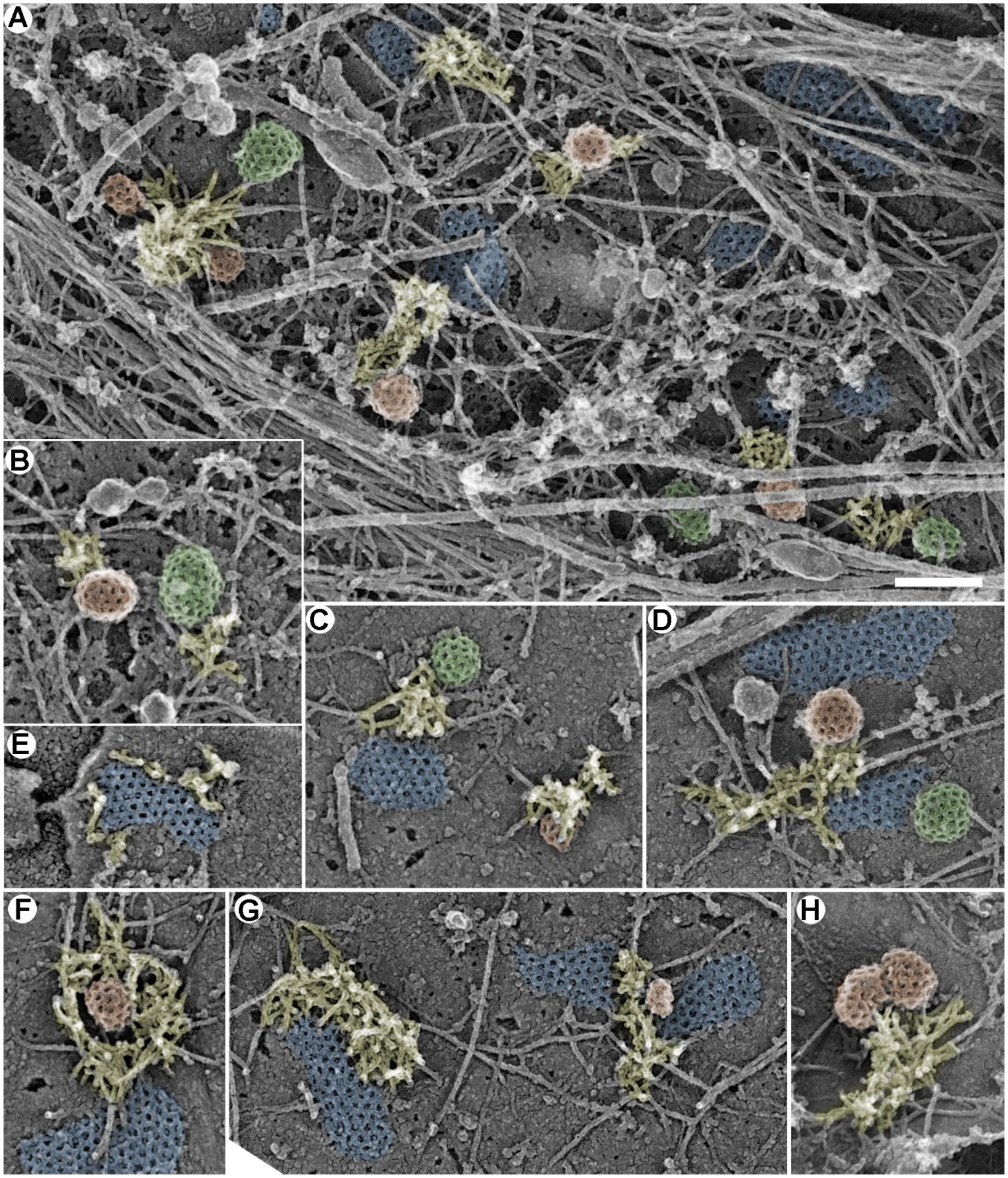

Supplementary Figure 2. CCS morphology in PtK2 cells. (A) A region of an unroofed cell showing flat (blue), dome-shaped (green) and spherical (orange) CCSs, some of which are associated with branched actin networks (yellow). (B-H) Examples of individual CCSs and associated branched actin networks. At spherical CCSs, branched actin network can form lateral comet tail-like structures (B, C, D, H) or surround the CCS (F). At flat CCSs, branched actin networks extend along the CCS perimeter (G, left) and sometimes appear to separate them from each other (G, right) or from dome-shaped (C, upper) or spherical (D) CCSs. In strongly unroofed samples (E), the remaining actin filament fragments (yellow) are attached along the perimeter of a flat CCS (blue), but not to the apical surface. Scale bar, 200 nm (refers to all panels).

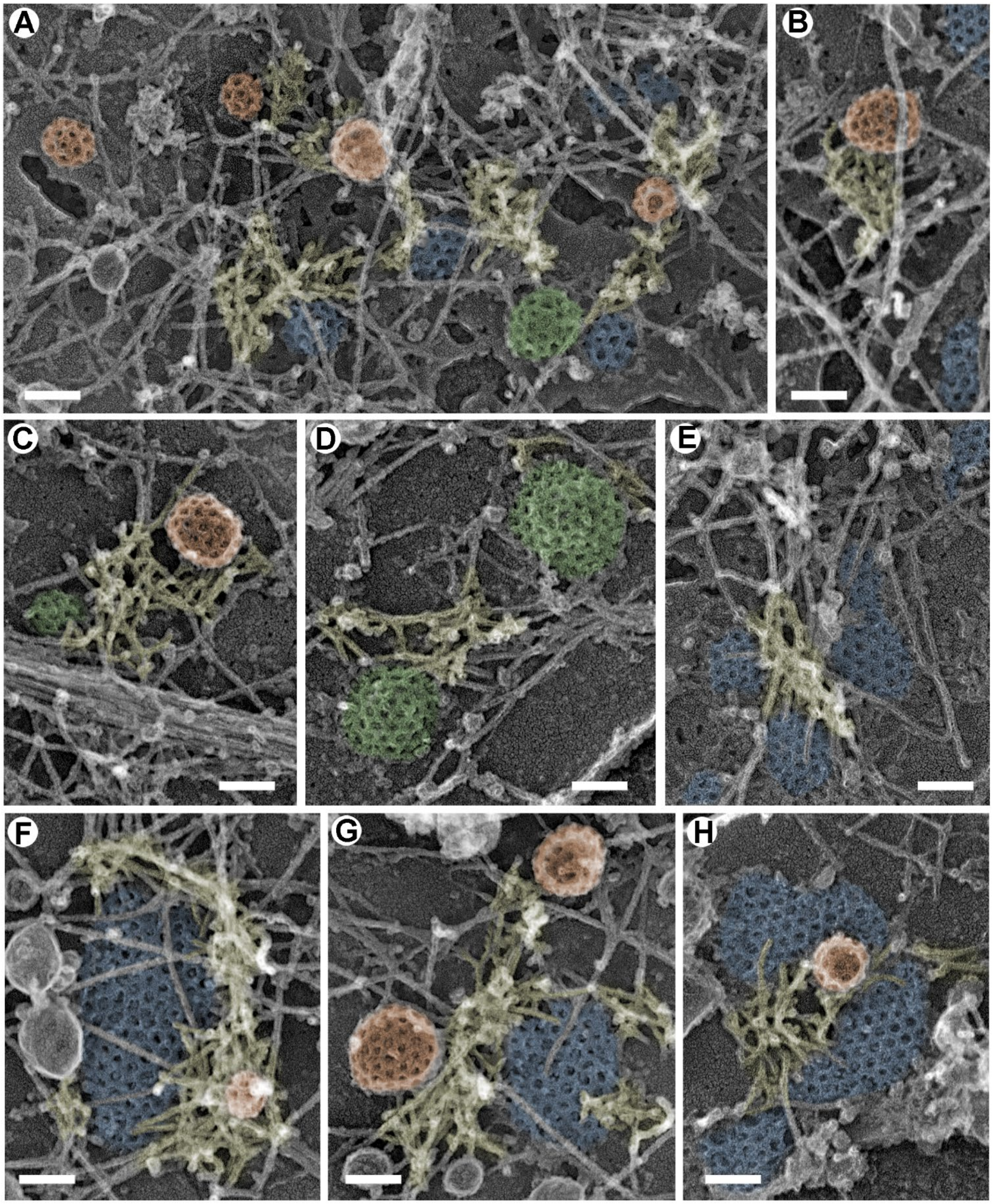

Supplementary Figure 3. CCS morphology in HeLa cells. (A) A region of an unroofed cell showing flat (blue), dome-shaped (green) and spherical (orange) CCSs, some of which are associated with branched actin networks (yellow). (B-H) Examples of individual CCSs and associated branched actin networks, which can form a comet tail-like structure at spherical (B, C, H) or dome-shaped (D, lower) CCSs. At flat CCSs, they extend along the CCS perimeter (F), localize between CCSs as if separating them from each other (E) or from spherical CCSs (G), and even apparently carve out a spherical CCS out of the interior of the flat CCS (H). Scale bars, 200 nm.

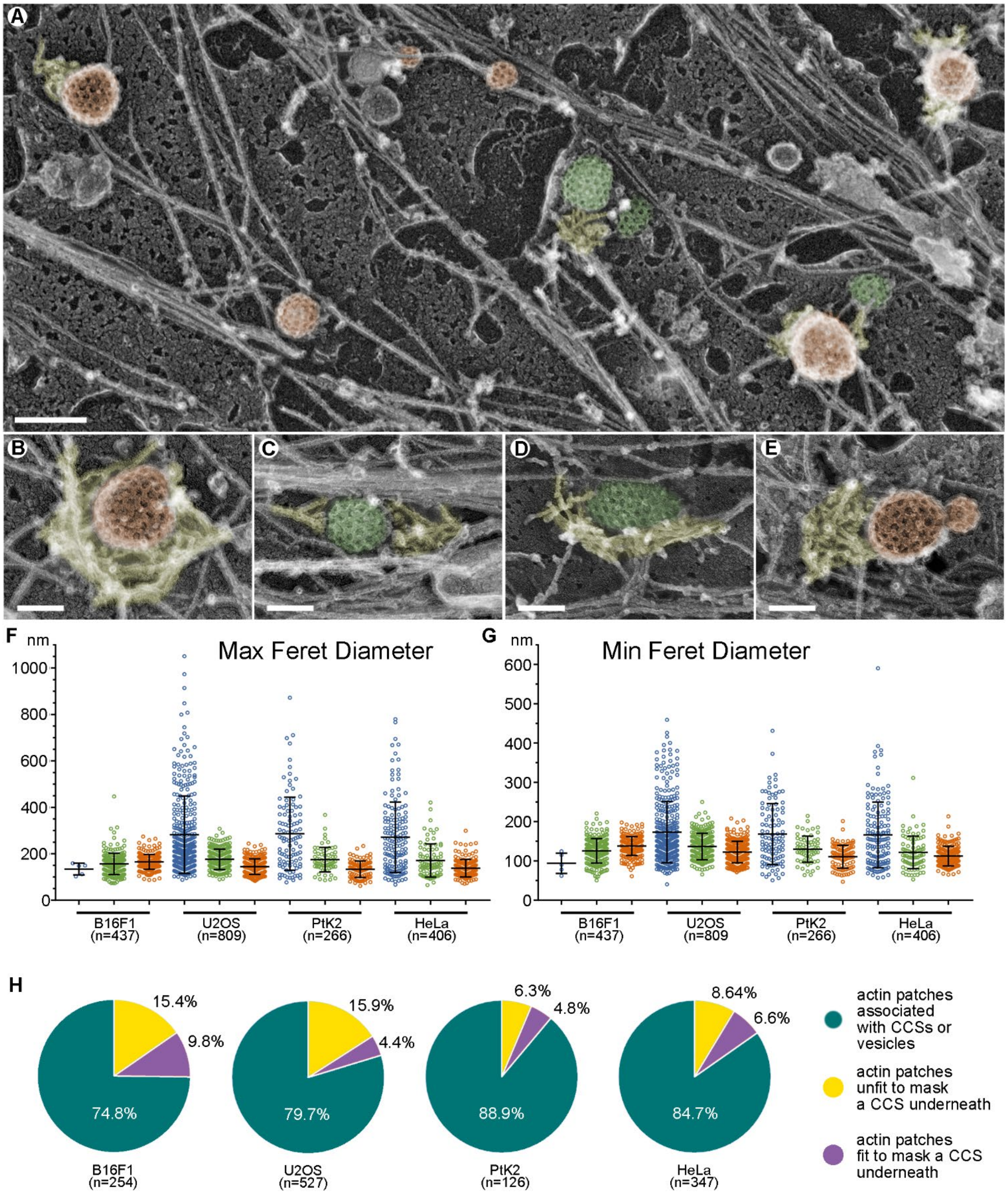

Supplementary Figure 4. (A-E) CCS morphology in B16F1 cells. (A) A region of an unroofed cell showing dome-shaped (green) and spherical (orange) CCSs, some of which are associated with branched actin networks (yellow). (B-E) Examples of individual CCSs and associated branched actin networks, which can form a lateral comet tail-like structure (E) or partially surround the CCS (B-D). Scale bars, 200 nm (A) and 100 nm (B-E). (F, G) Maximal (F) and minimal (G) Feret diameters of CCSs in PREM samples for indicated cell types. Error bars, mean±SD. (H) Categories of branched actin patches in unroofed cells. Only a very small subset of actin patches in each cell type has appropriate dimensions and density to be able to conceal a CCS underneath.

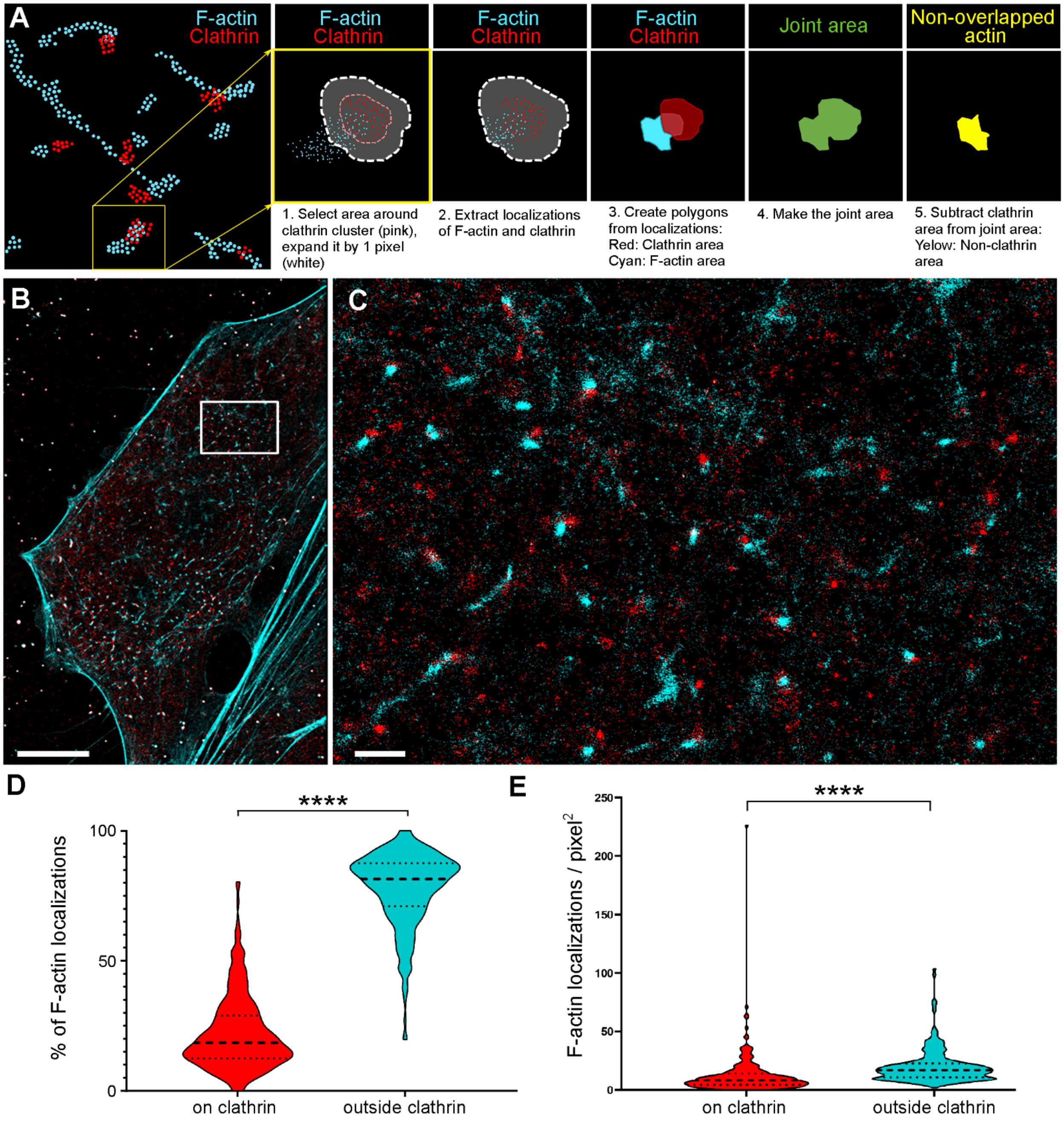

Supplementary Figure 5. STORM imaging of intact Ptk2 cells labeled with AlexaFluor488 phalloidin (cyan) and AlexaFluor405-647 antibody against CHC (red). (A) Workflow of colocalization analysis (see Methods for additional details). (B) Ptk2 cell imaged with STORM (same as in Figure 2P). (C) Enlargement of the boxed region in A depicting CCSs (red) with laterally associated F-actin patches (cyan). Scale bars, 10 µm (B) and 500 nm (C). (D) Percentages of actin localizations co-localized with clathrin clusters ("on clathrin") or localized in immediate vicinity of clathrin clusters ("outside clathrin"); ****, $p<0.0001$. (E) Densities of actin localizations co-localized with clathrin clusters ("on clathrin") or found in actin clusters in immediate vicinity of clathrin clusters ("outside clathrin"). For D and E, dash lines show medians, dotted lines show quartiles; ****, $p<0.0001$ (two-tailed Mann Whitney test); n= 10 cells, 268 ROIs and 5752 clathrin clusters from two independent experiments.

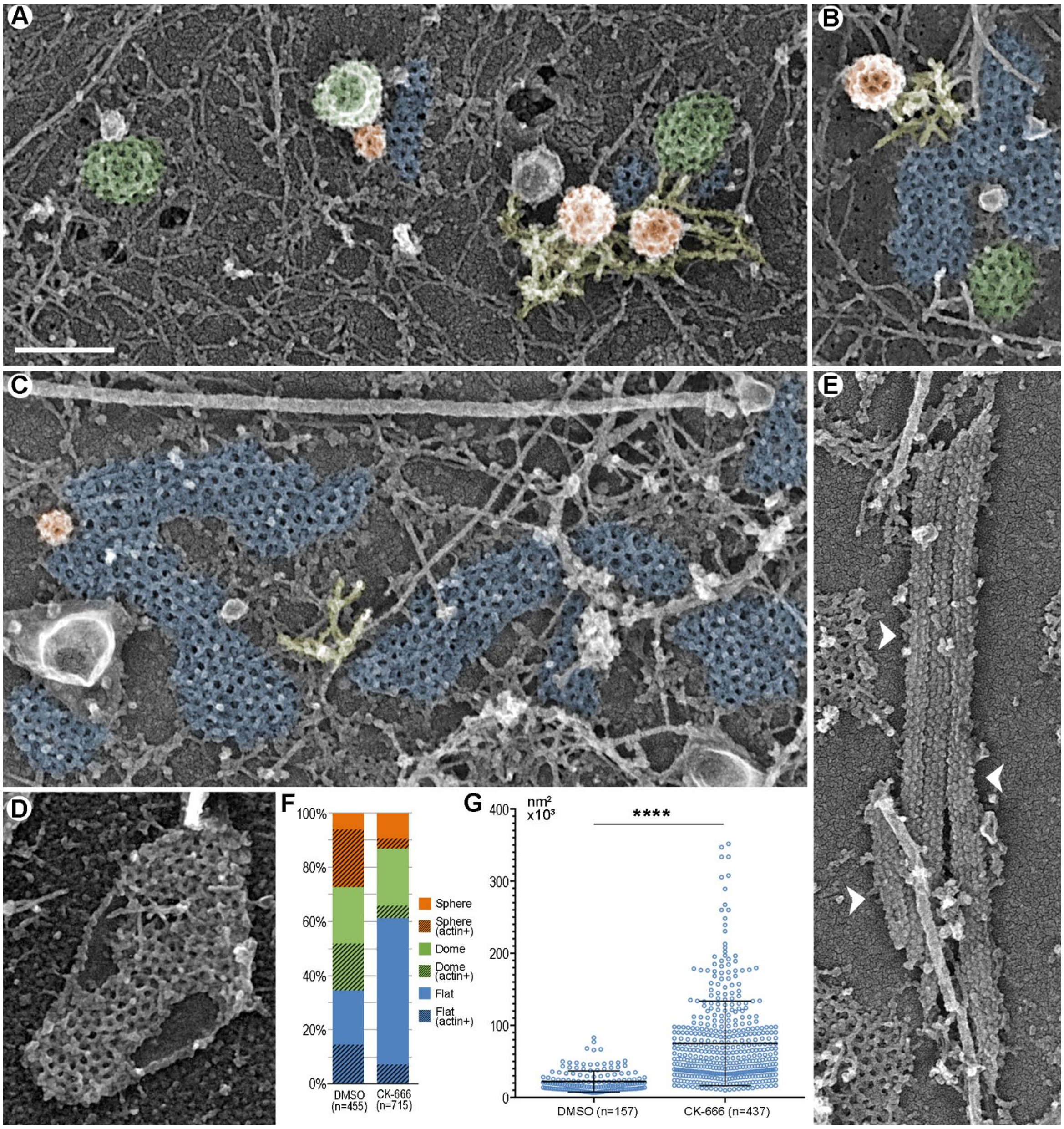

Supplementary Figure 6. Inhibition of the Arp2/3 complex by 200 µM CK-666 in U2OS cells increases occurrence and areas of flat CCSs at the expense of other CCS categories. (A) Control U2OS cells cultured in the presence of 0.1% FBS and DMSO exhibit flat (blue), dome-shaped (green) and spherical (orange) CCS, some of which are associated with branched actin networks (yellow). (B-E) Cells treated with CK-666 in the presence of 0.1% FBS contain abundant flat CCSs (blue) and infrequent spherical (orange) CCSs; small patches of uninhibited branched actin networks (yellow) can be found occasionally. (D) In stringently unroofed cells, flat CCSs are efficiently preserved due to adhesion. (E) Linear beaded aggregates formed in CK-666-treated cells (arrowheads). Scale bar, 200 nm (refers to all panels). (F) Percentages of different CCS shape categories in U2OS cells in indicated conditions. Hatched colors indicate a fraction of CCSs associated with branched actin networks within a given category. CK-666 treatment increases the percentage of flat CCSs and decreases the frequency of CCS association with branched actin networks. (G) Projection area of flat CCSs in indicated conditions. Error bars, mean ± SD; ****, $p<0.0001$; two-tailed Mann-Whitney test.

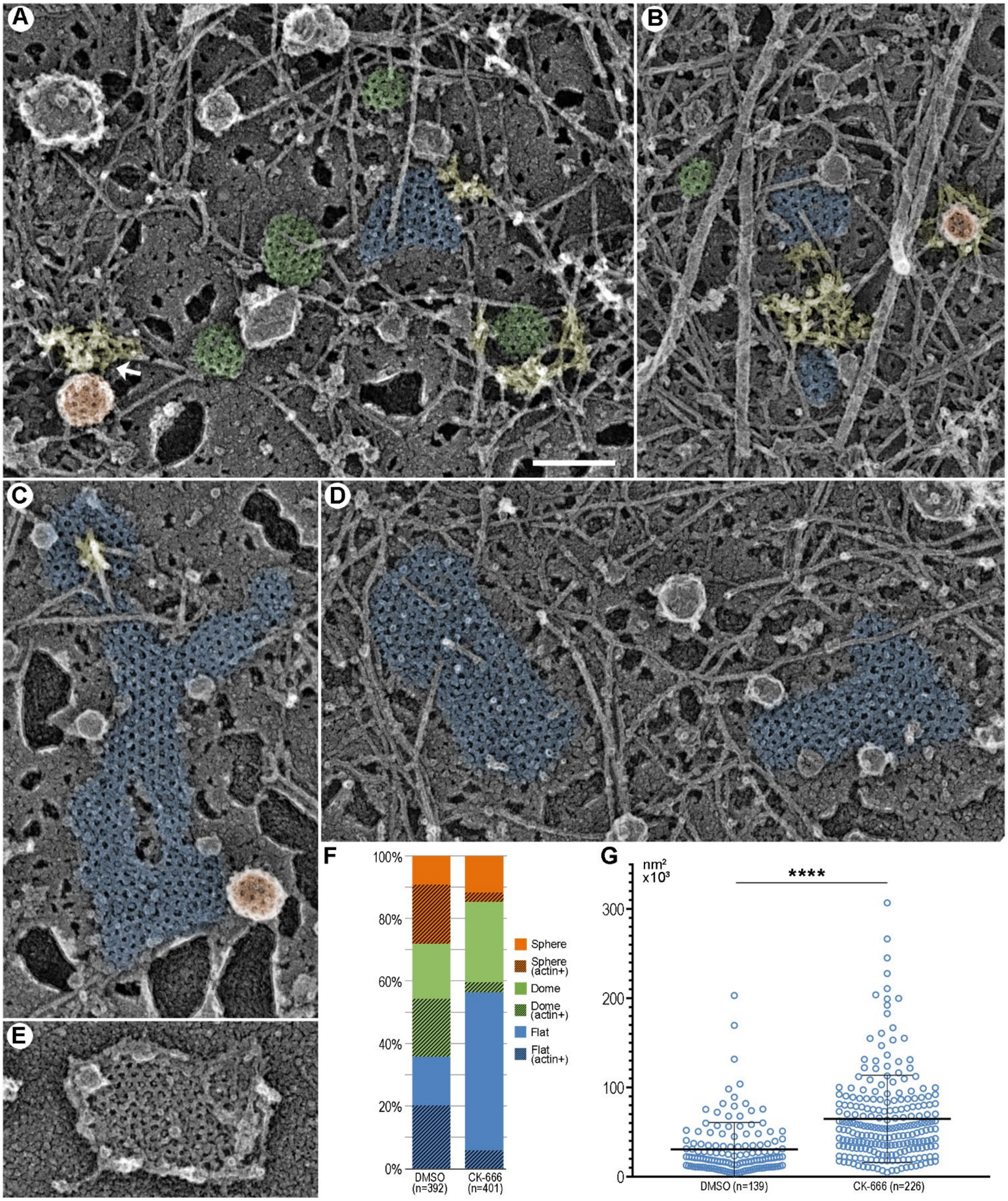

Supplementary Figure 7. Inhibition of the Arp2/3 complex by 200 μM CK-666 in HeLa cells increases occurrence and areas of flat CCSs at the expense of other CCS categories. (A, B) Control HeLa cells cultured in the presence of 0.1% FBS and DMSO exhibit flat (blue), dome-shaped (green) and spherical (orange) CCS, some of which are associated with branched actin networks (yellow). (C-E) Cells treated with CK-666 in the presence of 0.1% FBS contain abundant flat CCSs (blue) occasionally associated with small remaining patches of branched

actin network (C, yellow). Scale bar, 200 nm (refers to all panels). (E) In stringently unroofed cells, flat CCSs are efficiently preserved due to adhesion. (F) Percentages of different CCS shape categories in HeLa cells in indicated conditions. Hatched colors indicate a fraction of CCSs associated with branched actin networks within a given category. CK-666 treatment increases the percentage of flat CCSs and decreases the frequency of CCS association with branched actin networks. (G) Projection area of flat CCSs in indicated conditions. Error bars, mean ± SD; ****, p<0.0001, two-tailed Mann-Whitney test.

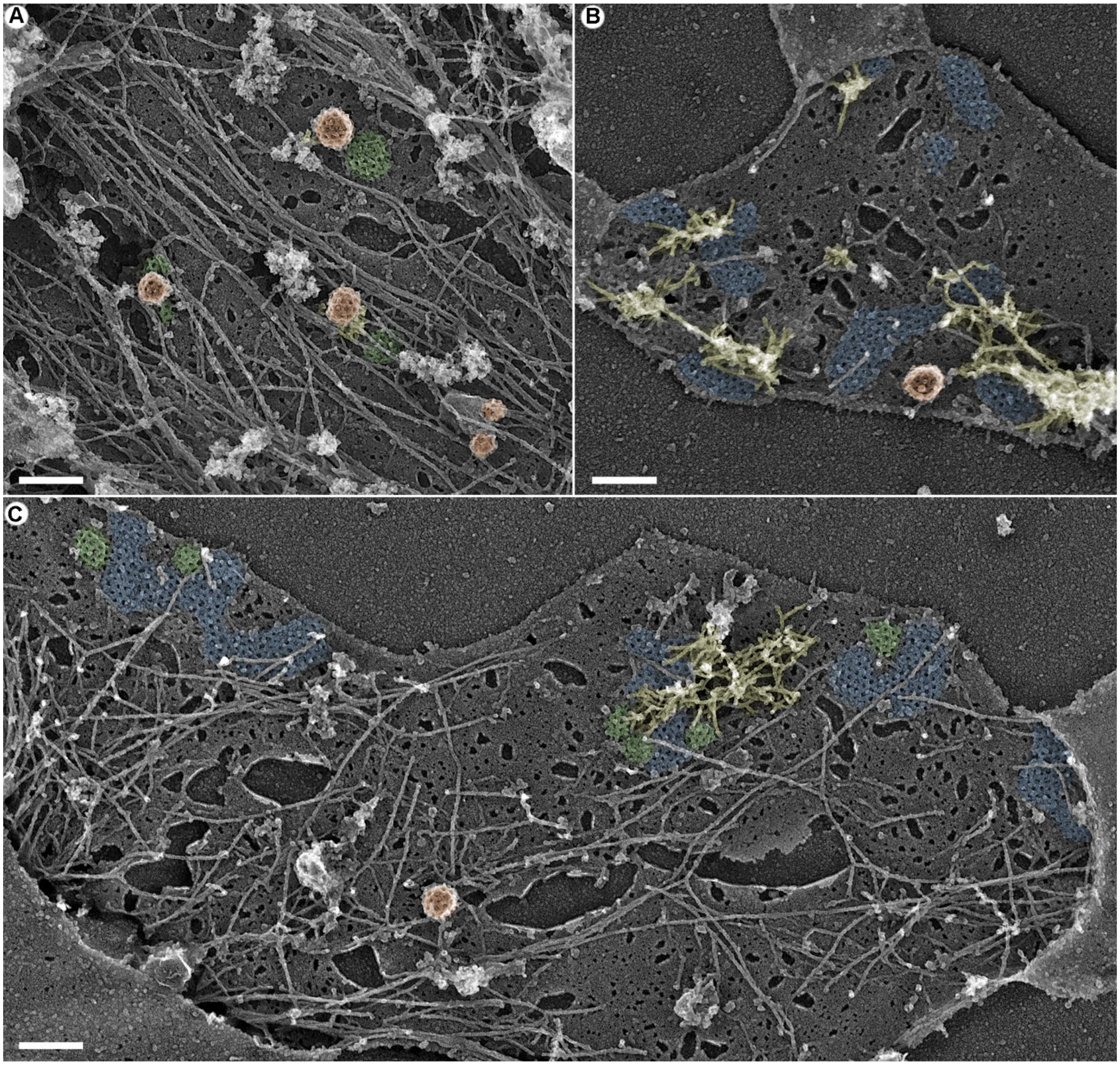

Supplementary Figure 8. Inhibition of the Arp2/3 complex in B16F1 cells leads to the formation of flat CCSs. (A) Control B16F1 cell treated with DMSO for 1 hour in the presence of 10% FBS exhibits dome-shaped (green) and spherical (orange) CCSs, some of which are associated with branched actin networks (yellow). (B, C) Cells treated with CK-666 in mild conditions (100 μM for 1 hour in the presence of 10% FBS) contain abundant flat CCSs (blue), as well as some dome-shaped (green) and spherical (orange) CCSs, associated with remaining patches of branched actin network (C, yellow). Scale bar, 200 nm (refers to all panels).
