# Supplementary material for: Actin polymerization promotes invagination of flat clathrin-coated lattices in mammalian cells by pushing at lattice edges
Source: Nat Commun. 2022 Oct 17;13:6127. doi: 10.1038/s41467-022-33852-2 (PMC9576739; doi:10.1038/s41467-022-33852-2)
Supplement: Supplementary file 3 — Description of Additional Supplementary Files [file 41467_2022_33852_MOESM3_ESM.pdf]

File name: Supplementary Movie 1

Description: Animation of tilted PREM images showing examples of 3D organization of actin-CCS association in U2OS cells. Color coding: Blue – flat CCSs; green – dome-shaped CCSs, orange – spherical CCSs; yellow – branched actin networks. Scale bars, 100 nm.

File name: Supplementary Movie 2

Description: Animation of tilted PREM images showing examples of 3D organization of actin-CCS association in PtK2 cells. Color coding: Blue – flat CCSs; green – dome-shaped CCSs, orange – spherical CCSs; yellow – branched actin networks. Scale bars, 100 nm.

File name: Supplementary Movie 3

Description: Animation of tilted PREM images showing examples of 3D organization of actin-CCS association in HeLa cells. Color coding: Blue – flat CCSs; green – dome-shaped CCSs, orange – spherical CCSs; yellow – branched actin networks. Scale bars, 100 nm.

File name: Supplementary Movie 4

Description: Animation of tilted PREM images showing examples of 3D organization of actin-CCS association in PtK2 cells treated with 200 µM CK-666 for 4 hours in the presence of 0.1% FBS. Color coding: Blue – flat CCSs; green – dome-shaped CCSs, orange – spherical CCSs; yellow – branched actin networks. Scale bars, 100 nm.

File name: Supplementary Movie 5

Description: Animation of tilted PREM images showing examples of 3D organization of actin-CCS association in PtK2 cells treated with DMSO for 4 hours in the presence of 0.1% FBS. Color coding: Blue – flat CCSs; green – dome-shaped CCSs, orange – spherical CCSs; yellow – branched actin networks. Scale bars, 100 nm.

File name: Supplementary Movie 6

Description: TIRF microscopy movie of endogenously expressed RFP-CLC in U2OS cells treated with either DMSO (left) or 200 µM CK-666 (right) for 4 hours before imaging. Time in min:sec.

File name: Supplementary Movie 7

Description: Animation of tilted PREM images showing examples of 3D organization of actin-CCS association in PtK2 cells treated with 200 µM CK-666 for 4 hours in the presence of 0.1% FBS and washed out for 30 sec. Color coding: Blue – flat CCSs; green – dome-shaped CCSs, orange – spherical CCSs; yellow – branched actin networks. Scale bars, 100 nm.

File name: Supplementary Movie 8

Description: Animation of tilted PREM images showing examples of 3D organization of actin-CCS association in PtK2 cells treated with 200 µM CK-666 for 4 hours in the presence of 0.1% FBS and washed out for 1 min. Color coding: Blue – flat CCSs; green – dome-shaped CCSs, orange – spherical CCSs; yellow – branched actin networks. Scale bars, 100 nm.

File name: Supplementary Movie 9

Description: Animation of tilted PREM images showing examples of 3D organization of actin-CCS association in PtK2 cells treated with 200 µM CK-666 for 4 hours in the presence of 0.1% FBS and washed out for 1.5 min. Color coding: Blue – flat CCSs; green – dome-shaped CCSs, orange – spherical CCSs; yellow – branched actin networks. Scale bars, 100 nm.

File name: Supplementary Movie 10

Description: Animation of tilted PREM images showing examples of 3D organization of actin-CCS association in PtK2 cells treated with 200 µM CK-666 for 4 hours in the presence of 0.1% FBS and washed out for 2 min. Color coding: Blue – flat CCSs; green – dome-shaped CCSs, orange – spherical CCSs; yellow – branched actin networks. Scale bars, 100 nm.

File name: Supplementary Movie 11

Description: TIRF microscopy movie of EGFP-CLC in epsin DKO MEFs treated either DMSO (left) or 100 µM CK-666 right) for 4 hrs before imaging. Time in min:sec.

File name: Supplementary Movie 12

Description: TIRF microscopy movie of EGFP-CLC in epsin TKO MEFs treated with either DMSO (left) or 100 µM CK-666 right) for 4 hrs before imaging. Time in min:sec.

File name: Supplementary Movie 13

Description: Animation of tilted PREM images showing examples of 3D organization of actin-microtubule association in the vicinity of CCSs in PtK2 cells. Color coding: Blue – flat CCSs; green – dome-shaped CCSs, orange – spherical CCSs; red – microtubules, yellow – branched actin networks apparently emerging from microtubules. Scale bars, 100 nm.
