# Supplementary material for: Actin polymerization promotes invagination of flat clathrin-coated lattices in mammalian cells by pushing at lattice edges
Source: Nat Commun. 2022 Oct 17;13:6127. doi: 10.1038/s41467-022-33852-2 (PMC9576739; doi:10.1038/s41467-022-33852-2)
Supplement: Supplementary file 17 — Reporting Summary [file 41467_2022_33852_MOESM17_ESM.pdf]

Corresponding author(s): Tatyana Svitkina

Last updated by author(s): Sep 21, 2022

Reporting Summary

Nature Portfolio wishes to improve the reproducibility of the work that we publish. This form provides structure for consistency and transparency in reporting. For further information on Nature Portfolio policies, see our Editorial Policies and the Editorial Policy Checklist.

Statistics

For all statistical analyses, confirm that the following items are present in the figure legend, table legend, main text, or Methods section.

n/a	Confirmed	
☐	☒	The exact sample size (*n*) for each experimental group/condition, given as a discrete number and unit of measurement
☐	☒	A statement on whether measurements were taken from distinct samples or whether the same sample was measured repeatedly
☐	☒	The statistical test(s) used AND whether they are one- or two-sided<br>*Only common tests should be described solely by name; describe more complex techniques in the Methods section.*
☒	☐	A description of all covariates tested
☐	☒	A description of any assumptions or corrections, such as tests of normality and adjustment for multiple comparisons
☐	☒	A full description of the statistical parameters including central tendency (e.g. means) or other basic estimates (e.g. regression coefficient) AND variation (e.g. standard deviation) or associated estimates of uncertainty (e.g. confidence intervals)
☐	☒	For null hypothesis testing, the test statistic (e.g. *F*, *t*, *r*) with confidence intervals, effect sizes, degrees of freedom and *P* value noted<br>*Give P values as exact values whenever suitable.*
☒	☐	For Bayesian analysis, information on the choice of priors and Markov chain Monte Carlo settings
☒	☐	For hierarchical and complex designs, identification of the appropriate level for tests and full reporting of outcomes
☒	☐	Estimates of effect sizes (e.g. Cohen's *d*, Pearson's *r*), indicating how they were calculated

Our web collection on statistics for biologists contains articles on many of the points above.

Software and code

Policy information about availability of computer code

Data collection	For PREM samples , images were acquired by an ORIUS 832.10W CCD camera driven by Gatan Digital Micrograph 1.8.4 software (Gatan, Warrendale, PA) . The original files in dm3 format were converted into tiff files using the same software. TIRF microscopy was performed using Eclipse TiE inverted microscope (Nikon) equipped with a CFI Apochromat TIRF 100 × 1.49 NA oil objective, iXon3 DU-897 back-illuminated EMCCD camera (Andor) and a perfect focus system driven by Nikon (NIS)-Elements Advanced Research software (Version 4.50). STORM super-resolution imaging was performed in Hilo (highly inclined laminated optical sheet) illumination mode using a Nanoimager S microscope equipped with a 100X 1.4 NA oil immersion objective and Hamamatsu Orca Flash 4 V3 sCMOS camera driven by Nanoimager™ Software (version 1.10.0.11025) from Oxford Nanoimaging. Subsequent image processing and figure preparation was done by Adobe Photoshop software (version 21.1.0 20200212.r.106 or 23.5.0 20220809.r.669 0e07917)
Data analysis	Image J (v1.53c, NIH) were used for quantification of morphometry of electron microscopy data and fluorescence images. Channel fine alignment of STORM images was performed with Matlab (Version R2021b, The MathWorks, Inc.). Statistical analyses were done using GraphPad Prism 9.0.0. software.

For manuscripts utilizing custom algorithms or software that are central to the research but not yet described in published literature, software must be made available to editors and reviewers. We strongly encourage code deposition in a community repository (e.g. GitHub). See the Nature Portfolio guidelines for submitting code & software for further information.

Data

Policy information about availability of data

All manuscripts must include a data availability statement. This statement should provide the following information, where applicable:

- Accession codes, unique identifiers, or web links for publicly available datasets
- A description of any restrictions on data availability
- For clinical datasets or third party data, please ensure that the statement adheres to our policy

The quantitative data generated in this study are provided in Source Data file. All other data supporting the findings of this study are available from the corresponding author on reasonable request. A MATLAB script for a fine alignment of STORM images have been deposited in the GitHub database [https://github.com/melikelakadamyali/ClusterOverlap].

Field-specific reporting

Please select the one below that is the best fit for your research. If you are not sure, read the appropriate sections before making your selection.

☒ Life sciences ☐ Behavioural & social sciences ☐ Ecological, evolutionary & environmental sciences

For a reference copy of the document with all sections, see nature.com/documents/nr-reporting-summary-flat.pdf

Life sciences study design

All studies must disclose on these points even when the disclosure is negative.

Sample size	No statistical method was used to predetermine sample size. Sample size was chosen based on previous experience and standards in the field (Nat Cell Biol, 2019, 21(5): 603-613; Nat Cell Biol, 2020, 22(6): 674-688; Nature Commun. 2020, 23, 11(1): 4818). Sample size is stated in the main text or figure legends, or indicated in the figures.
Data exclusions	No EM data were excluded from analyses except for technically unsuccessful experiments (e.g. unsatisfactory exposure of the structures of interest in the cell interior). For the quantification of TIRF microscopy images, two PtK2 cells treated with CK-666, in which the procedure produced an ROI covering nearly the entire cell, were excluded from quantification.
Replication	Repetitive experiments (at least twice) have been done to confirm consistency of results. Hundreds of individual examples from many cells and experiments were examined in PREM experiments to account for individual biological variations.
Randomization	No randomization was applied as there were no human or animal subjects used in this study. The samples were organized according to cell types and treatment conditions and the order of analysis does not influence the experimental outcomes.
Blinding	Data collection and analysis was not performed blinded, as there were no human or animal subjects in this study. Blinding was not considered also based upon experience and similar published studies. All experiments were repeated at least twice and included a large amount of cells to ensure reproducibility,

Reporting for specific materials, systems and methods

We require information from authors about some types of materials, experimental systems and methods used in many studies. Here, indicate whether each material, system or method listed is relevant to your study. If you are not sure if a list item applies to your research, read the appropriate section before selecting a response.

Materials & experimental systems

n/a	Involved in the study	
☐	☒	Antibodies
☐	☒	Eukaryotic cell lines
☒	☐	Palaeontology and archaeology
☒	☐	Animals and other organisms
☒	☐	Human research participants
☒	☐	Clinical data
☒	☐	Dual use research of concern

Methods

n/a	Involved in the study	
☒	☐	ChIP-seq
☒	☐	Flow cytometry
☒	☐	MRI-based neuroimaging

Antibodies

Antibodies used	Mouse monoclonal X-22 clathrin heavy chain antibody (#Ab2731) used at 1:1000 in 5% BSA in PBS was from Abcam. Alexa Fluor 594 goat anti-mouse IgG secondary antibody (#A11032) from Thermo Fisher Scientific. Secondary goat-anti-mouse IgG (115-0050062) for the custom labeling with Alexa Fluor 405-Alexa Fluor A647 activator/reporter dye pair was from Jackson ImmunoResearch labs.

Validation

The Mouse monoclonal X-22 clathrin heavy chain antibody (#Ab2731) has been validated and detailed information could be found on the website from the manufactures as listed: https://www.abcam.com/clathrin-heavy-chain-antibody-x22-ab2731.htm. In addition, this antibody has been used in our previously publication: Collins et al., 2011, Curr Biol 21, 1167-1175, and it has been also used in multiple publications, including Nat Cell Biol , 22, 1357–1370 (2020).

Eukaryotic cell lines

Policy information about cell lines

Cell line source(s)

Ptk2 cells were from ATCC. Genome-edited U2OS cells and HeLa cells, both endogenously expressing RFP-tagged clathrin light chain A (CLTA) and EGFP-tagged dynamin2 (DNM2), were from Dr. David Drubin (Nat Cell Biol, 13, 331-337, 2011 and J Cell Biol, 205, 721-735, 2014). B16F1 cells were from Dr. C. Ballestrem (J Cell Biol 160, 409-421, 2003); they were originally from Dr. GL. Nicolson (J Natl Cancer Inst 56(2); 285-91, 1976). Conditional epsin DKO MEFs with deleted epsin2 and epsin3 genes and floxed epsin1 gene were from Dr. Pietro De Camilli (Elife, e03311, 2014).

Authentication

Cell lines were not authenticated by ourselves.

Mycoplasma contamination

Cell lines were regularly tested for mycoplasma contamination by DAPI staining and were found negative.

Commonly misidentified lines
(See ICLAC register)

No commonly misidentified cell line was used.

March 2021
